# Long-term impacts of captivity on skull morphology and endocranial volume in a marsupial carnivore

**DOI:** 10.1098/rsos.240868

**Published:** 2025-05-21

**Authors:** Emily L. Scicluna, Marissa L. Parrott, Leila Siciliano-Martina, Margot Michaud, Kylie Robert, Peter T. Green

**Affiliations:** ^1^ Department of Ecology, Environment and Evolution, La Trobe University, Melbourne, Victoria, Australia; ^2^ La Trobe University Research Centre for Future Landscapes, Melbourne, Victoria, Australia; ^3^ School of Biosciences, The University of Melbourne, Melbourne, Victoria, Australia; ^4^ Wildlife Conservation and Science, Zoos Victoria, Parkville, Victoria, Australia; ^5^ Department of Biology, Texas State University, San Marcos, TX, USA; ^6^ Evolution and Diversity Dynamics Lab, University of Liege, Liege, Belgium; ^7^ Université de la Guyane, Cayenne, French Guiana

**Keywords:** morphometrics, conservation, phenotypic change, cranial morphology, reintroduction biology, captive husbandry

## Abstract

A challenge in modern conservation is maintaining ecological roles and natural behaviours of wildlife in an anthropogenic world. Captive breeding has been linked to morphological changes that may impact individual fitness for reintroduction to the wild. Changes in skull morphology and brain size may be linked to functional and behavioural changes, influencing survival outcomes. These issues have been explored in numerous eutherian mammals, but rarely in metatherians. We compared skull morphology and endocranial volume in a carnivorous marsupial, the fat-tailed dunnart (*Sminthopsis crassicaudata*), between wild-derived and captive individuals maintained in a university laboratory colony over tens of generations. Skulls from captive dunnarts were brachycephalic, with significantly shorter basal and upper molar lengths, broader zygomatic widths and longer palate and toothrow lengths, compared with wild counterparts. Captive-bred dunnarts also had a mean endocranial volume 3.8% larger than wild individuals. These traits relate to dietary, cognitive and sensory capabilities and can be linked to functional differences within captive and wild populations. Therefore, changes to these regions could have substantial fitness consequences in natural habitats. By addressing the ways laboratory management can influence morphological traits, we can reassess broader captive management techniques to improve the success of future breeding and reintroduction programmes.

## 1. Introduction

Long-term insurance populations and captive breeding programmes form the foundations of many conservation efforts for endangered species globally [1,2]. Captive management also provides a valuable opportunity for rapid intervention and species recovery when *in situ* population management is not possible [3,4]. However, a potential disadvantage is an unintended phenotypic change that may occur in captivity [5–7]. Most research in this field has focused on behavioural changes, with some species showing a loss of predator avoidance or variation to defensive [8], reproductive [9] or territorial behaviours, poor hunting skills [6,10,11] and heritable changes in temperament that may affect reintroduction success (e.g. [12]). Increasingly, however, the focus is shifting towards potential morphological consequences of captivity [7,13,14], including body size and shape [15], skull morphology [16–18], brain size [19], limb development and proportion [20,21] and internal morphology [22]. Changes in morphological traits related to brain size and skull shape can have strong consequences for functionality and can therefore modify an animal’s ability to survive and reproduce when introduced to a natural habitat [7]. Patterns of skull change have been documented in many carnivorous eutherian mammals (Order Carnivora), where captive animals can show changes in skull morphology and brain size, but the impact of captivity on carnivorous marsupials (Order Dasyuromorphia) is virtually unknown (but see [23]). More research is needed to better inform the captive management of carnivorous marsupials for conservation purposes. While the animals in this study were not managed for *ex situ* conservation, laboratory-bred fat-tailed dunnarts provide an opportunity to observe and learn from possible changes from long-term captivity.

Changes in skull dimensions are well documented in some species maintained in captivity [16]. Black-footed ferrets (*Mustela nigripes*), for example, were among the first endangered species bred in captivity, following removal from the last wild population in Wyoming in 1985−1986. Wisely *et al*. [24] documented changes in skull morphology after >10 years of captive breeding, including a significant size decrease (3–10%) compared with wild individuals. Antonelli *et al*. [25] compared 23 skull measurements in black-footed ferrets and found that 65% of measurements were significantly different between long-term captive-bred and wild ferrets. Similarly, Siciliano-Martina *et al*. [16] found a significant difference in the size and shape of Mexican wolf (*Canis lupus baileyi*) skulls, with captive-bred wolves showing greater shape variation, but smaller overall skulls, compared with wild counterparts. In both cases, the morphological differentiation was attributed to the small founding populations, along with the relatively ‘soft’ texture of the diets in captivity. Masticatory musculature is tightly correlated with dietary texture, where ‘tough’ food items require enhanced cranial muscles, which are reflected by the relative length and width of skull structures [26,27]. Therefore, providing diets that are less mechanically demanding can influence skull shapes, leading to morphological differentiation between captive and wild populations [14,28,29].

Captive populations can also display differences in brain sizes [30]. These differences have been linked to dietary availability, especially where captive animals receive high-nutrient diets that may be less variable than what is available in the wild. On an individual level, brain size development is due, in part, to nutritional quality, where individuals who are deprived of high-quality diets may develop smaller brains [31]. Therefore, animals whose wild diets are highly stochastic may develop larger brain sizes in captivity where high-quality diets are consistently provided. However, captivity is also associated with smaller brain sizes in some species, especially in domesticated laboratory or agricultural animals [32,33], a trait that has been linked to depauperate cognitive environments experienced by the animals. Conversely, no brain size changes have been found in studies of captive-bred marsupials, in which naturalistic diets and high levels of environmental enrichment were provided (M.L.P. 2024, unpublished data) [23]. Despite the divergent outcomes of these studies, there is some evidence to suggest that captivity can affect brain volume.

Although the consequences of morphological changes for reintroduction success have not been tested empirically, there is growing awareness that such changes may be disadvantageous because they decouple internal and external cranial traits, altering skull functionality [16,17,34–36]. When captive mammals develop modified morphological traits associated with behaviours necessary for survival (e.g. jaw structure that assists specific feeding mechanisms [37]), it may limit their functionality and have adverse effects upon reintroduction to the wild. However, the effects of morphological changes on reintroduction success remain unknown until after the animals have been released, by which stage any consequences may be irreversible [13]. This can have large implications for population management and wildlife reintroductions, which is particularly concerning given the urgent need for intervention to avoid further species extinctions [7].

Our study examines changes in skull measurements and endocranial volume (a useful proxy for brain size) in a locally threatened species, the carnivorous marsupial, fat-tailed dunnart (*Sminthopsis crassicaudata*). We compared skulls from captive-bred individuals from a long-term laboratory colony with skulls from wild individuals (museum specimens), to assess whether captivity had significant effects on skull dimensions and endocranial volume. Given the novelty and limitations of the captive diet provided, we expected to find a morphological difference between captive and wild dunnart populations. In particular, we expected cranial regions associated with masticatory musculature (e.g. zygomatic width) to be wider in captive individuals, given the relatively soft texture of the captive diet, and for brain size to be smaller in the captive laboratory-based environment, as this trend has been observed in numerous other carnivores of various taxa (for example, see [14,35]).

While laboratory management can be considered an extreme form of captivity, our findings should inform broader husbandry management approaches. In laboratories, this may include selection of reproductive individuals based on desired morphological traits (i.e. selective breeding), while in conservation programmes, this could be the importance of providing environmental enrichment and diets that mimic the nutritional quality and hardness of wild food items to maintain natural skull morphology.

## 2. Material and methods

### 2.1. Study species and sources of skull material

The fat-tailed dunnart is a small (15–20 g) carnivorous marsupial (family Dasyuridae) that occurs in grasslands and semi-arid regions of southern Australia [38]. The species predominantly feeds on invertebrates (e.g. spiders, beetles and centipedes), but also occasionally small vertebrates (e.g. house mouse (*Mus musculus*) and small reptiles) [39,40]. Morphological variations in coat colour, body size and ear and tail length were historically used to describe subspecies (e.g. [41]), but while Cooper *et al*. [42] established the existence of two genetically distinct populations (evolutionarily significant units (ESUs)) these did not suport the classification of subspecies. The fat-tailed dunnart was listed as a threatened species in Victoria, Australia, as of 2023 [43] on the basis of population decline [44,45]. The species has been maintained in numerous long-term captive populations across Australia to study various aspects of its reproductive biology and physiology (e.g. [46–48]).

Captive skulls were collected from animals that had died of natural causes or were culled as part of colony maintenance at LTU between 2015 and 2018. Individuals ranged between 166 and 1412 days old. The LTU captive population was established in 2001 with founders from the University of Adelaide (UoA). The origins of that captive population are documented back to the mid-1960s with wild-caught progenitors from the Northern Territory, Queensland and South Australia [49], a geographic range that encompassed both ESUs identified by Cooper *et al*. [42]. The captive dunnarts used in this study had been bred at LTU for 12−18 generations, but given the UoA history, the lineage has been in captivity for tens of generations. Both institutions incorporated frequent genetic outcrossing, often swapping individuals between other captive institutions and with occasional wild genetic influx. Because of this uncertainty, the number of generations in captivity has not been used as a covariate in our analyses. Captive LTU skulls were prepared using dermestid beetles [50], and only adult skulls of known sex and age, and with no apparent or known pathology to the animal, were considered for analysis. Museum skulls of wild dunnarts encompassed the geographic ranges of the two major mtDNA haplotype clades [42] to match the probable origins of the LTU colony. Only adult specimens (determined by full eruption of M^1–4^ as per [51]) with complete information for sex and collection location were included.

Captive individuals were housed individually (except when maintained in breeding groups) or when females had dependent young and fed ad libitum a prepared diet of dry cat food (Whiskas Adult beef and lamb), soaked in water overnight then mixed with wet cat food (Whiskas Adult Jellymeat loaf) and calcium carbonate [45]. The diet was supplemented three times per week with live invertebrates (mealworms, *Tenebrio molitor,* and crickets, *Acheta domestica*). Vitamins (Penta-vite oral liquid) were added to water and provided ad libitum in laboratory drink bottles.

### 2.2. Skull morphology

**Figure 1.**
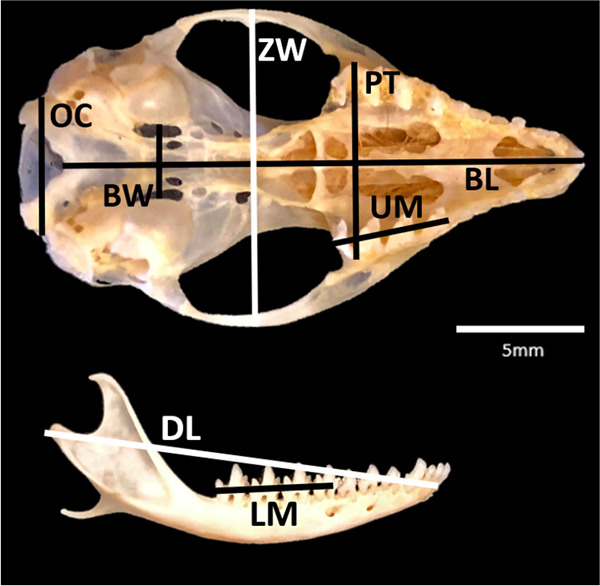
Morphological measurements obtained from fat-tailed dunnart skulls, excluding endocranial capacity. BL, basal length; ZW, zygomatic width; BW, inside bulla width; OC, occipital condyle width; UM, upper molars (m^1–4^); LM, lower molars (m^1–4^); PT, palate and toothrows; DL, dentary length.

We measured skull traits from a total of 201 individuals, comprising 108 captive-bred (64 females and 44 males) and 93 wild-derived specimens (39 females and 54 males). We measured eight standard skull and dental traits: basal length (BL), zygomatic width (ZW), inside bulla width (BW), occipital condyle (OC) width, upper molar (UM) length, lower molar (LM) length, palate and toothrows (PT) length and mandibular dentary length (DL; figure 1). All measurements were taken to the nearest 0.01 mm using digital callipers by one researcher (E.L.S.) to minimize observer error. A magnifying glass and light were used to assist visualization and to ensure measurements were taken accurately. Percentage measurement error between measurements was <1%, with an average of 0.56% (Appendix 1). UM and LM lengths were taken on the right side of skull/mandible, and in the absence of this (e.g. missing teeth), the measurement was excluded.

### 2.3. Endocranial volume

Necropsies of freshly deceased, captive individuals (*n* = 12) revealed that the fat-tailed dunnart brain fills the entire cranial cavity [45]. Therefore, endocranial volume is likely to be a suitable surrogate measure for fat-tailed dunnart brain volume, as documented in numerous species (e.g. [52,53]). Endocranial volume was measured by filling each skull with size 12 lead shot via the foramen magnum [23,52,54], with continuous gentle tapping to ensure compaction of the lead shot. Once the cavity was full, the shot was weighed to the nearest 0.01 g. Measurement error was reduced to below 1.5% by repeated measurement (five times by one operator, E.L.S.) for each skull, falling within the range of variation of other studies using the same technique [23,54,55].

To calculate endocranial volume from lead shot mass, a calibration curve was created to establish the relationship between lead shot volume and mass [23]. Using a measuring cylinder for volume, mass (g) was recorded for each 0.1 ml increment between 1 and 4 ml, with five independent replicates of volume and mass per increment. Replicated regression [56] was used to derive the relationship between lead shot mass and volume (ml) = (0.1604 × lead shot (g)) + 0.105 (*r*^2^ = 0.999, *p* < 0.001), to ensure that the recorded mass provided highly accurate volume measurements.

### 2.4. Fontanelle measurements

**Figure 2.**
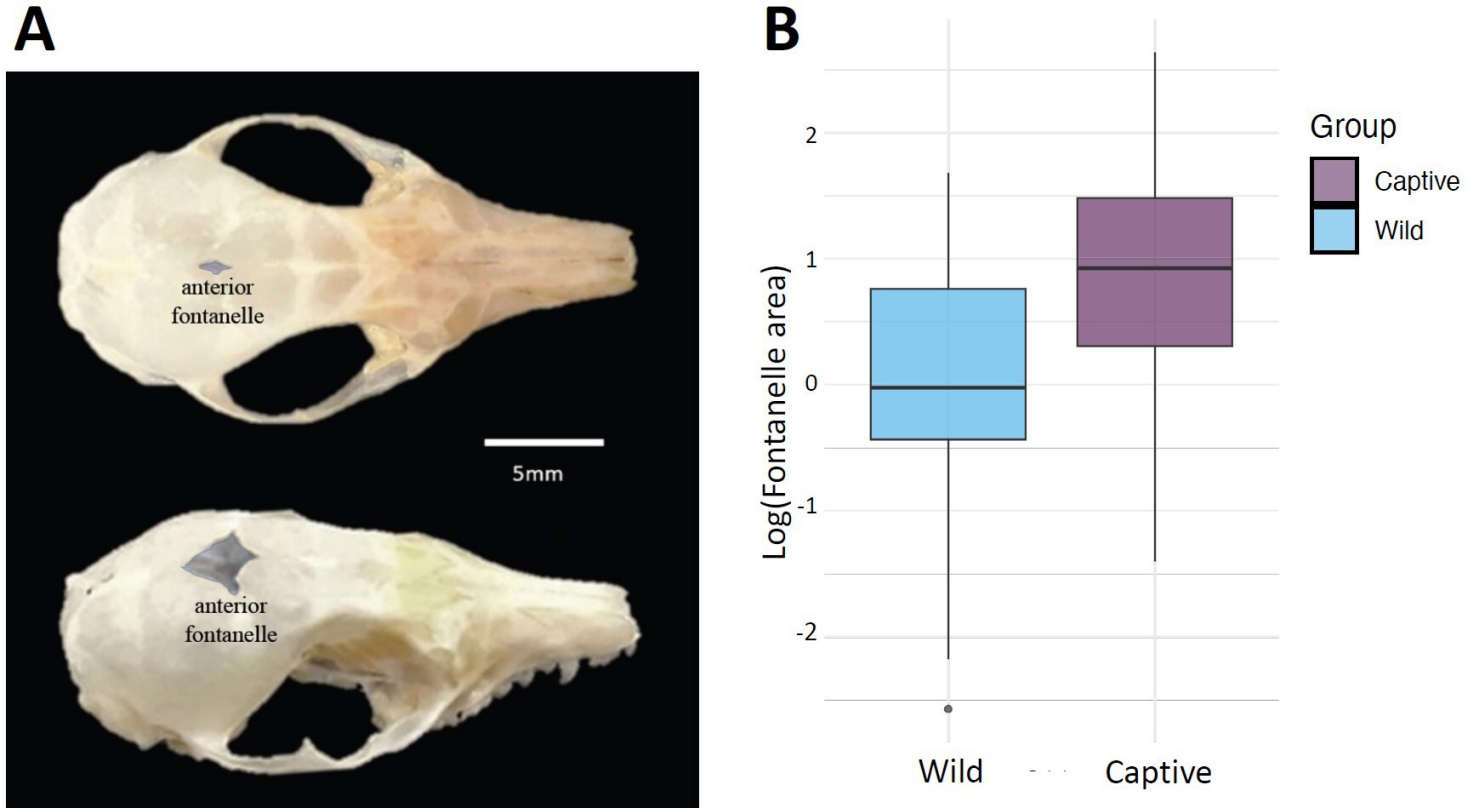
Fontanelle area in captive and wild fat-tailed dunnarts. (A) Dorsal view of two captive *S. crassicaudata* skulls, illustrating variation in fontanelle size. (B) Boxplot comparing log-transformed fontanelle area between captive (purple) and wild (blue) specimens.

Fontanelles represent cranial sutures where two segments of bone remain separated through infant development, acting to accommodate brain growth inside the skull [57]. Presence of these in adult skulls is unusual, so we measured the length and width of these features to the nearest 0.1 mm along the sagittal and coronal sutures, respectively, and because in almost all cases they were diamond-shaped, we calculated the area as 0.5 × length × width (figure 2).

### 2.5. Statistical analysis—univariate analyses

We evaluated the eight linear measures and endocranial volume across populations (captive/wild) and sexes (female/male), including their interaction. To account for individual size variation, we applied a log transformation to all measures [58,59]. Despite this transformation, the data remained non-normally distributed and violated the assumption of homogeneity of variances. To address this, we conducted non-parametric aligned rank transformation (ART) analyses and extracted standard effect sizes (partial eta-squared; *η*^2^) using the *ArtTool* package [60,61] in R v. 4.2.3 [62].

We also evaluated the occurrence of skull fontanelles between populations (captive and wild) using a chi-squared test for independence. We also evaluated the relative area of the fontanelles between populations and sexes using Welch’s unpaired *t*-tests.

### 2.6. Statistical analysis—multivariate analyses

To evaluate multivariate trait space, we performed a principal components (PCs) analysis on the log-transformed linear measures. To determine whether trait variation differed between populations, we conducted a permutation test for multivariate homogeneity of group dispersions using 1000 permutations. We extracted PC1 and PC2 as independent shape estimates, and, given their non-normal distribution and violation of homogeneity of variances, we performed ART analyses to assess the difference across populations (captive/wild), sexes (female/male) and their interaction. To further quantify the morphological divergence between captive and wild, we conducted linear discriminant functional analysis (LDFA) using the log-transformed linear measures. From this analysis, we conducted a multivariate analysis of variance (MANOVA) and extracted Wilks’s lambda to evaluate how well population origin (captive or wild) distinguished between groups, and Mahalanobis distance to quantify the degree of morphological separation.

## 3. Results

### 3.1. Univariate analyses

Captive and wild populations of fat-tailed dunnarts differed significantly in four of eight skull measurements, plus endocranial volume (table 1). Captivity had a large negative effect on BL (*p* < 0.001, *η*^2^ = 0.12) and a moderate negative effect on UM length (*p* < 0.05, *η*^2^ = 0.02). However, captivity had a moderately positive effect on ZW (*p* < 0.01, *η*^2^ = 0.04) and PT length (*p* < 0.01, *η*^2^ = 0.04). There was evidence of an interaction between population and sex for LM length (*p* < 0.05, *η*^2^ = 0.03) and mandibular DL (*p* < 0.01, *η*^2^ = 0.04). Examination of group LM means suggests that while both sexes of wild animals (5.5 ± 0.3 mm) and captive females (5.5 ± 0.2 mm) had similarly sized LM dimensions, captive males differed, with slightly shorter LM dimensions (5.4 ± 0.2 mm). There was strong evidence for an interaction between population and sex for DL (*p* < 0.01, *η*^2^ = 0.04). An examination of group means indicates that although there was no overall difference between captive and wild individuals, captivity had approximately equal but opposite effects depending on sex, with a shortening of mean DL in male dunnarts (by 0.4 mm) and a lengthening in females (0.4 mm).

**Table 1.** Summary statistics and results of the univariate non-parametric ART analyses comparing morphological measurements, endocranial volume and fontanelle area of captive and wild fat-tailed dunnarts. Summary statistics include the mean, s.d. and sample size (*n*) for each population origin (captive or wild) and sex (female and male). The ART analyses evaluated the effects of population origin, sex and their interaction. Results are reported as *F*-statistics (*F*), with significant differences indicated by bold *p* values (*p*). Standard effect sizes are reported as partial eta-squared (*η*^2^). Trait measures include BL, basal length; ZW, zygomatic width; BW, bulla width; OC, occipital condyle; UM, upper molars; LM, lower molars; PT, palate and toothrows; DL, dentary length; Vol, endocranial volume; Font, fontanelle area.

	summary statistics								analysis								
	population				sex				population			sex			interaction		
	captive		wild		female		male		*F*	*p*	*η* ^2^	*F*	*p*	*η* ^2^	*F*	*p*	*η* ^2^
	mean ± s.d.	*n*	mean ± s.d.	*n*	mean ± s.d.	*n*	mean ± s.d.	*n*									
**BL** (**mm**)	22.7 ± 0.8	94	23.6 ± 1.5	77	23.0 ± 1.1	87	23.2 ± 1.4	84	23.41	**<0.001**	0.12	0.40	>0.05	2.0 × 10^−3^	3.27	>0.05	0.02
**ZW** (**mm**)	13.5 ± 0.4	103	13.2 ± 0.8	80	13.3 ± 0.6	92	13.4 ± 0.6	91	6.79	**<0.01**	0.04	0.19	>0.05	1.0 × 10^−3^	0.63	>0.05	4.0 × 10^−3^
**BW** (**mm**)	3.0 ± 0.3	95	3.1 ± 0.4	75	3.1 ± 0.3	86	3.0 ± 0.3	84	0.53	>0.05	2.0 × 10^−3^	1.04	>0.05	6.0 × 10^−3^	0.73	>0.05	1.0 × 10^−3^
**OC** (**mm**)	5.9 ± 0.3	94	5.9 ± 0.3	75	5.9 ± 0.2	85	5.9 ± 0.3	84	1.72	>0.05	1.03 × 10^−2^	3.59	>0.05	0.02	1.0 × 10^−3^	>0.05	1.1 × 10^−5^
**UM (M^1–4^) (mm**)	5.0 ± 0.2	100	5.1 ± 0.2	88	5.0 ± 0.2	93	5.1 ± 0.2	95	4.61	**<0.05**	0.02	0.04	>0.05	2.0 × 10^−4^	0.36	>0.05	2.0 × 10^−3^
**LM (M^1–4^) (mm**)	5.5 ± 0.2	103	5.5 ± 0.3	87	5.5 ± 0.2	96	5.5 ± 0.3	94	0.01	>0.05	5.5 × 10^−5^	3.34	>0.05	0.02	5.44	**<0.05**	0.03
**PT** (**mm**)	8.1 ± 0.3	104	7.9 ± 0.4	88	8.1 ± 0.4	97	8.0 ± 0.4	95	8.35	**<0.01**	0.04	8.35	**<0.01**	0.04	1.12	>0.05	6.0 × 10^−3^
**DL** (**mm**)	18.4 ± 0.7	106	18.4 ± 1.1	87	18.4 ± 0.9	98	18.4 ± 0.9	95	0.72	>0.05	3.8 × 10^−3^	4.0 × 10^−3^	>0.05	2.4 × 10^−6^	8.79	**<0.01**	0.04
**Vol** (**mm^3^**)	466.1 ± 20.2	90	449.0 ± 44.2	65	448.7 ± 31.3	81	469.9 ± 32.5	74	8.81	**<0.01**	0.05	19.82	**<0.001**	0.12	0.35	>0.05	2.0 × 10^−3^

Captivity had a large positive effect on endocranial volume (table 1; population main effect *p* < 0.01, *η*^2^ = 0.05) that affected both sexes equally (population × sex interaction *p* > 0.05, *η*^2^ = 2.0 × 10^−3^), with males having larger brain cases overall (sex main effect *p* < 0.001, *η*^2^ = 0.12). On average, endocranial volume was 3.8% larger in captive dunnarts (466.1 ± 20.2 mm^3^) than their wild conspecifics (449.0 ± 44.2 mm^3^; table 1).

We also detected differences in the population-level variation for some of the metrics. Levene’s tests for homogeneity of variances that compared captive and wild populations showed the captive group was significantly less variable than the wild group for six skull dimensions (BL, ZW, BW, LM, PT and DL) and endocranial volume (*p* < 0.05), but equally variable for OC and UM (see Appendix 2).

**Figure 3.**
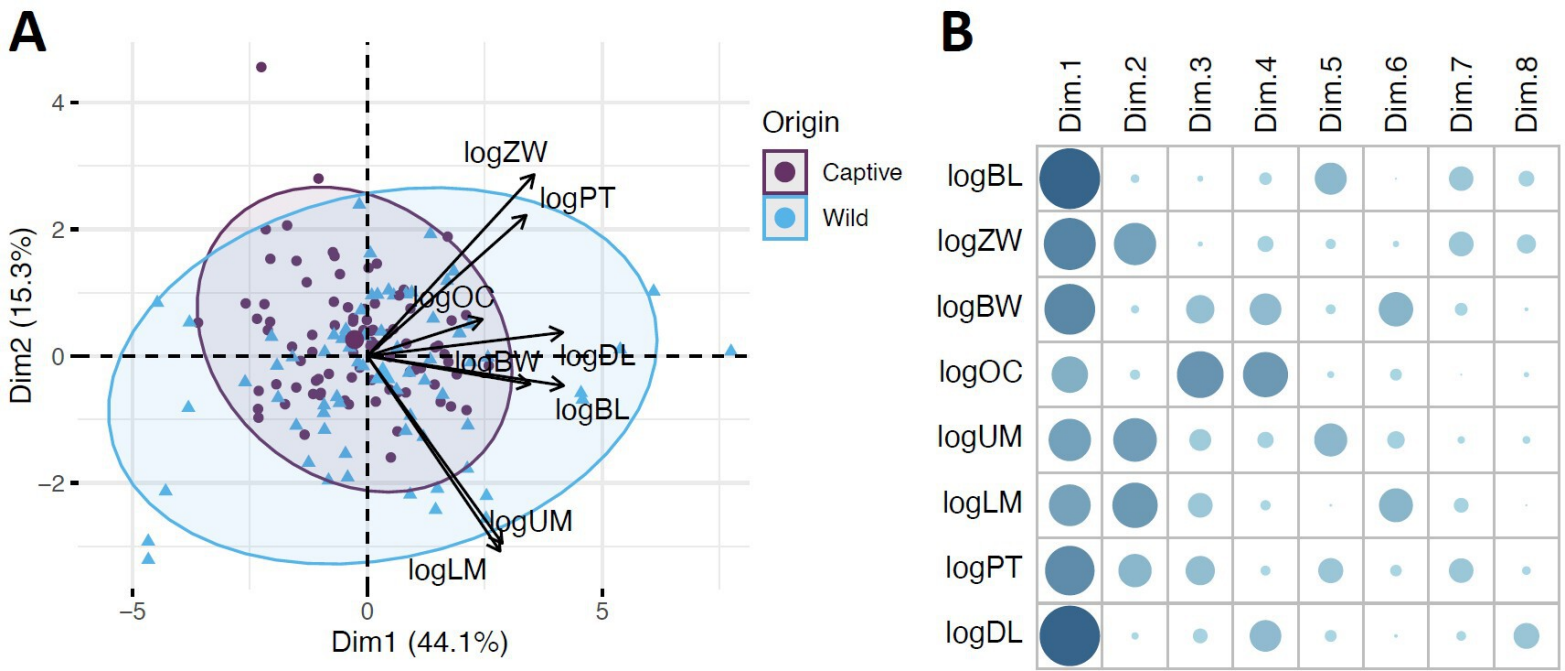
Multivariate trait space of captive and wild fat-tailed dunnarts. (A) PC biplot showing PC1 (Dim 1) and PC2 (Dim 2). Arrows indicate the primary traits shaping different regions of trait space. Dark purple points represent captive specimens, while light blue points represent wild specimens. Ellipses denote 95% confidence intervals for each population. (B) Correlation plot illustrating trait contributions to the PCs. Larger, darker dots indicate a greater contribution to a given PC.

Captive and wild populations differed in the incidence of an anterior fontanelle, where the frontal and parietal cranial bones had not fused leaving a hole in the skull where the sagittal and coronal sutures meet (figure 3). Fontanelles were more than twice as common in captive-bred animals (78% of 116) than in wild animals (32% of 68 animals; *χ*^2^_1184_ =33.754, *p* < 0.001). Fontanelle area of captive individuals ranged from 1.0 to 7.34 mm (sagittal suture) and 0.45 to 4.31 mm (coronal suture), while wild animals ranged from 0.61 to 4.03 mm at the sagittal suture and 0.16 to 2.6 mm at the coronal suture. The mean area of the fontanelle was greater in captive dunnarts (3.2 ± 2.6 mm^2^; *n* = 89) than in wild dunnarts (1.5 ± 1.3 mm^2^; *n* = 27). Welch’s two-sample *t*-tests did not detect a significant difference in log-transformed fontanelle area between males and females (*t* = −0.56, *p* > 0.05). However, a significant difference was detected between wild and captive populations (*t* = 3.97, *p* < 0.001; figure 3).

### 3.2. Multivariate analysis

**Figure 4.**
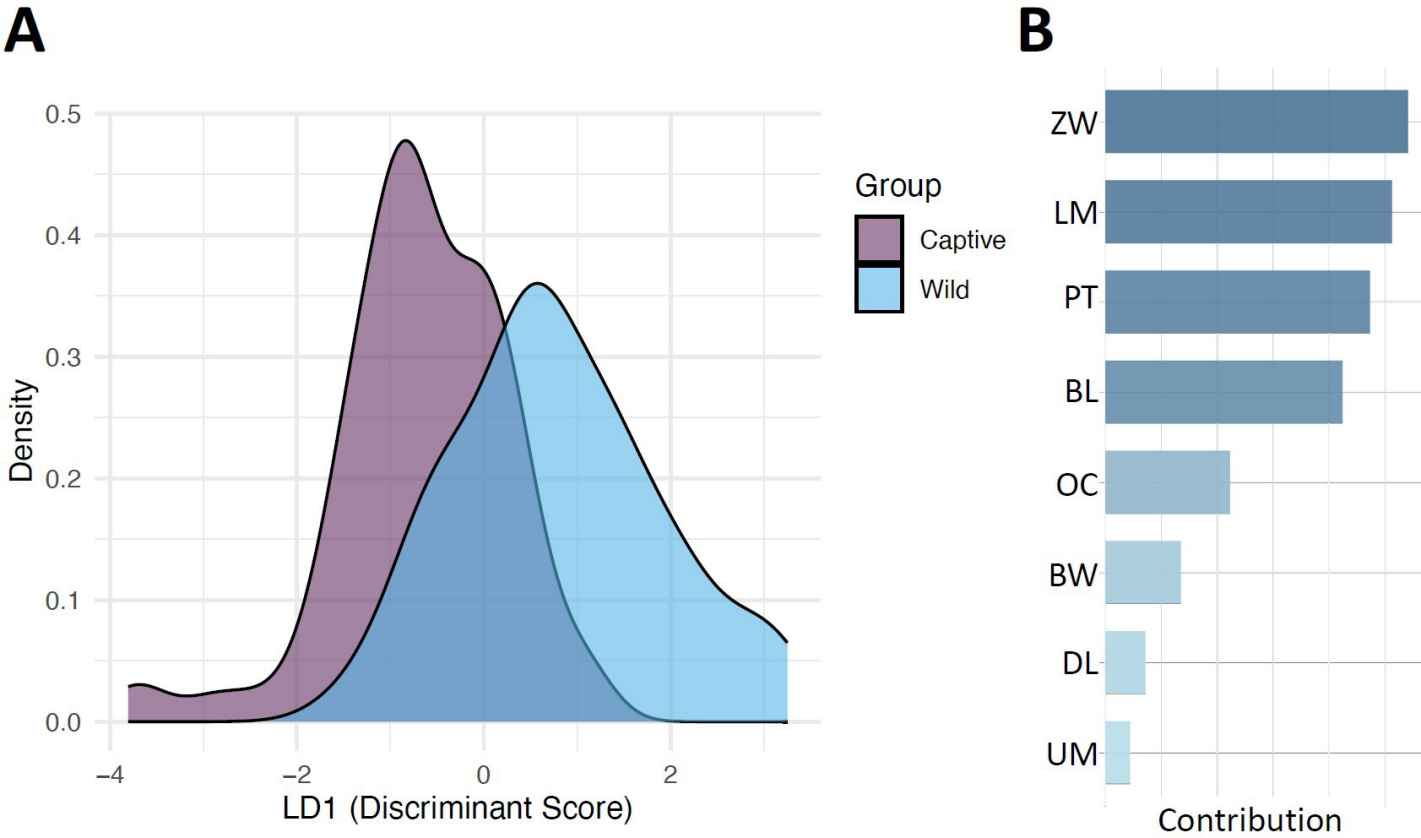
Differentiation between captive and wild fat-tailed dunnarts based on LDFA. (A) Density plot of linear discriminant function 1 (LD1) scores illustrating group separation between captive (purple) and wild (light blue) specimens. Higher density regions indicate where most specimens fall along the LD1 axis, while non-overlapping areas highlight differentiation between groups. (B) Trait contributions to LD1. Bar lengths indicate the absolute magnitude of each trait’s contribution, with darker colours indicating stronger effects. Traits are ordered by their importance in distinguishing between captive and wild populations.

PC1 accounted for roughly 44% of the total variation and was primarily driven by BL, DL, ZW and BW. PC2 explained roughly 15% of the total variation, with the strongest contributions from ZW, LM and UM (figure 4). ART analysis revealed a significant difference between captive and wild populations for PC1 (*p* < 0.05, *η*^2^ = 0.03) and PC2 (*p* < 0.01, *η*^2^ = 0.05). However, no significant difference was found between sexes or the interaction of population and sex (table 2). The permutation test for multivariate homogeneity of group dispersions indicated significantly greater trait variation in wild dunnarts difference in trait variation between populations (*F* = 20.63, *p* < 0.001), with wild dunnarts displaying greater morphological variations.

**Table 2.** ART analyses of PC1 and PC2 evaluated the effects of population origin, sex and their interaction. Results are reported as *F*-statistics (*F*), with significant differences indicated by bold *p* values (*p*). Standard effect sizes are reported as partial eta-squared (*η*^2^).

	analysis								
	population			sex			interaction		
	*F*	*p*	*η* ^2^	*F*	*p*	*η* ^2^	*F*	*p*	*η* ^2^
**PC1**	4.46	**<0.05**	0.03	0.15	>0.05	1.0 × 10^−3^	0.42	>0.05	3.0 × 10^−3^
**PC2**	7.11	**<0.01**	0.05	0.17	>0.05	1.0 × 10^−3^	0.02	>0.05	1.0 × 10^−4^

LDFA correctly classified 78.57% of specimens based on their multivariate trait profiles, with ZW, LM, PT and BL contributing most to group differentiation. MANOVA revealed a significant effect of population origin on multivariate trait space (Wilks’s lambda = 0.65, *F* = 9.61, *p* < 0.001), supporting morphological differentiation between captive and wild populations. Additionally, the Mahalanobis distance between captive and wild group centroids was 0.35, further reinforcing divergence in multivariate trait space. These results suggest that captive and wild dunnarts exhibit distinct morphological variations, with population origin influencing overall skull shape.

## 4. Discussion

Captive-bred fat-tailed dunnarts had significantly shorter, wider skulls and a larger mean endocranial volume than wild dunnarts. While previous studies have recorded shortening and widening of carnivore skulls in captivity (e.g. [14,35]), to our knowledge this is the first-ever record of increased endocranial volume in a captive marsupial. This is significant because detectable morphological change may be linked to behavioural changes or less obvious phenotypic changes that render individuals less fit for wild habitats. In this instance, these changes may be linked to dietary, cognitive and sensory capabilities, indicating the potential for functional differences between captive and wild individuals. These results can be used as a case study for long-term captive individuals intended for eventual wild reintroduction, as morphological changes may influence survival outcomes.

### 4.1. The effects of captivity on skull dimensions

Numerous studies (e.g. [7,16,19]) have reported differential effects of captive environments on mammalian skulls, with some dimensions undergoing significant change while others remain unaffected. We found that captive individuals displayed a significantly shorter BL and UM length (M^1–4^) and a wider ZW and PT length (table 1), although the mandible (as measured by mandibular DL) was not affected. This suggests that captive dunnarts had shorter muzzles, demonstrating a more brachycephalic skull compared with their wild counterparts. When combined, these changes in skull dimensions are reflected in the overall differences between wild and captive individuals in the multivariate analysis (figure 3). While sex explained some differences in particular dimensions (i.e. PT; table 1), the overall difference between wild and captive populations did not depend on sex.

There are several potential explanations for why the skulls of the captive fat-tailed dunnarts differed from their wild counterparts. First, the mechanical properties of their food would be markedly different. Fat-tailed dunnarts are carnivorous and in the wild, feed predominantly on an array of invertebrates (spiders, beetles, centipedes, etc) and occasionally small vertebrates such as frogs and reptiles [39,40]. It is ethically challenging and (sometimes) prohibitively costly to provide an identical-to-wild diet in a laboratory setting, which can often consist of hundreds of individuals to maintain outbred status. Instead, captive fat-tailed dunnarts were fed a much softer diet of pre-prepared mixes supplemented with a limited variety of harder-bodied, live invertebrates. A similar diet is provided across many other laboratory dunnart colonies [48,63,64], though the effects on skull morphology have not been investigated. Some of the captive fat-tailed dunnart skulls appeared to have calcification around the teeth, further evidence of a predominantly softer diet compared with wild counterparts, leading to increased calculus build-up [65–67]. Notably, the captive-bred individuals used in this study ranged from 166 to 1412 days old, while wild fat-tailed dunnarts have an average lifespan of approximately 547 days (1.5 years) [68]. Hence, although this difference in age between the sample groups is unlikely to influence skull morphology, dramatic differences in age would almost certainly influence tooth condition [69].

We detected shorter, wider skulls associated with the captive fat-tailed dunnarts, reflecting similar changes documented in captive lions [70,71], black-footed ferrets [25] and coyotes [35]. Many studies have concluded that the mechanical properties of food can affect skull morphology [14,25,28,37,72–74]. For example, Hollister [70,71] recorded increased ZW and shortening of the skull (BL) of captive lions (*Panthera leo*) compared with their wild counterparts. He suggested that this was caused by the jaw masseter muscles exerting less pressure on the brain case, resulting in decreased lateral compression. Cooper *et al*. [28] also recorded different shapes (but not size) in captive lions and tigers compared to wild, and this also was linked to mechanical differences in diet. Mandibular morphology is also associated with musculature and the mechanical properties of food [75]; therefore, it may not be surprising that we found UM (M^1–4^) and PT lengths were significantly reduced. Future studies could measure additional morphological measurements, particularly surrounding jaw function, temporalis musculature and biomechanical differences.

The nutritional quality and composition of food, particularly vitamins and minerals that affect bone development, may also cause morphological changes in the skulls of captive individuals. For example, Cordy [76] recorded cranial thickening in captive subadult baboons (*Papio* sp.) due to abnormal ratios of calcium and phosphorus, and permanent deformity of the mandible was recorded in dromedary camels (*Camelus dromedarius*) due to insufficient protein and calcium intake [77]. Similarly, vitamin D deficiency led to rickets in captive-born chimpanzees (*Pan troglodytes*) that were raised indoors under skylights and fed only milk, but this was corrected with injections of slow-release vitamin D [78].

Stereotypic behaviours, which are more commonly observed in captive environments than in the wild [79], may have further influenced the skull shape of captive fat-tailed dunnarts in this study. Stereotypic behaviours involve repetitive movements, such as pacing, circling or over-grooming [79], and therefore involve repetitive muscle usage. Some individuals in this study were particularly prone to excessive grooming or running in tight circles in their enclosures (E. L. S & K. R 2019, personal observation). Species most prone to stereotypies in captive environments are those with large home ranges [80] or highly specialized diets that are often challenging to replicate in captivity [81]. The home range size of fat-tailed dunnarts is not known, but they have been recorded travelling at least 600 m [68], which is considerable for an animal weighing approximately 15 g. Other dunnart species have relatively large home ranges for their size. For example, the Julia Creek dunnart (*Sminthopsis douglasi*) has a home range of 8 ha and weighs approximately 55 g [82], the sandhill dunnart (*Sminthopsis psammophila*) has a home range of 7.8 ha and weighs approximately 34 g [83] and the white-footed dunnart (*Sminthopsis leucopus*) has a home range of 1 ha and weighs approximately 23 g [84]. Given the likelihood that fat-tailed dunnarts have similarly large home ranges and travel similar distances, they may be more likely to exhibit stereotypic behaviour in captivity due to the simplification of their habitat (cf. [16]). This may be particularly prevalent in animals housed in laboratory settings, with smaller enclosures (e.g. laboratory rodent boxes), fewer enclosure furnishings and fewer opportunities for social interaction or enrichment than in outdoor or modern conservation breeding programmes.

### 4.2. The effects of captivity on endocranial volume

The mean endocranial volume of captive fat-tailed dunnarts is larger than that of wild animals, which is at odds with a plethora of other studies showing a decrease in either brain size or endocranial volume associated with both domestication and captive breeding programmes (e.g. [30] and references therein). The simplest explanation for why captive dunnarts had slightly larger endocranial volumes is that in captivity, dunnarts grow to larger average body size (mass) than their wild counterparts. There is some evidence this is the case and that it could account for the increase in endocranial volume documented in this study (see Appendix 3).

Conversely, endocranial volume may be influenced by changes in the thickness of skull bones in captive populations. The skulls of captive animals were noticeably thinner than wild skulls, often to the point of translucence. It is plausible that even if the outer dimensions of the braincase do not vary between wild and captive populations, endocranial volumes may be larger in captive-bred individuals due to bone thinning in captivity.

Endocranial volume was less variable among captive individuals than in wild-derived animals. Given the relationship between the brain sizes of wild and domesticated conspecifics, in which domestic animals often have significantly smaller brains due to selection for docility, absence of predation pressure and easily acquired resources [85], we might expect greater variability in the brain sizes of captive breeding populations. However, equally variable brain sizes have been documented in wild and captive breeding populations of mink [86], waterfowl [52] and cavies/guineapigs [87]. In our study, reduced variation among captive animals can likely be explained by diet. Captive animals were all fed the same diet that was uniform in both its mechanical and nutritional properties, so any effects of diet on skull morphology would be approximately equal among individuals. Conversely, the wild individuals were collected from sites across much of the geographic range of the species, including both arid and temperate sites, and encompassing a range of habitats. Environmental variation fosters trait diversity among species in both plants and animals [88], and there is evidence that the hardness of invertebrates eaten by wild dunnarts varies significantly between arid and temperate locations [89]. The greater variability among the skulls of wild individuals likely reflects the diversity of habitats and climatic zones from which the specimens were collected.

### 4.3. Incidence of the anterior fontanelle

Another consequence of captivity for fat-tailed dunnarts was the increased incidence of anterior fontanelles; 78% of captive animals featured the presence of an open fontanelle, more than twice the incidence in wild animals (33%; figure 2). The mean size of the fontanelle in captive animals was about twice the size of that in wild animals. While persistent fontanelles have been noted in some mammalian taxa (e.g. [90]), they are usually regarded as a neonatal characteristic, where the cranial bones have not fully ossified due to immature physiological development [57,91]. Although ossification of the neurocranium occurs later in marsupials than in other mammals due to the rudimentary state of foetal development at birth (e.g. [92,93]), it is unlikely that the high incidence of fontanelles in the captive sample was due to age. The adult fat-tailed dunnarts used in this study ranged between 166 and 1412 days old, much older than the age at which ossification of the skull with complete closure of the fontanelles occurs in other marsupials such as the northern brown bandicoot *Isoodon macrourus* (25 days [94]), the common brushtail possum *Trichosurus vulpecula* (49 days; [94]) or the Virginia opossum *Didelphis virginiana* (28 days [95]). These individuals also showed fully erupted adult teeth, another morphological indicator of adulthood [24,51,96]. It is therefore reasonable to expect that all captive animals used in the study should have displayed fully ossified skulls inclusive of closed fontanelles.

In humans, delayed fontanelle closure has been attributed to a variety of skeletal disorders [97–101]. Chromosomal abnormalities and genetic disorders [102] or inbreeding [103] are other possible reasons for this morphological abnormality. The greater incidence of persistent fontanelles in the captive population of fat-tailed dunnarts may be linked to issues such as inbreeding. While there was genetic exchange with other colonies when possible, these animals had also been captive-bred for tens of generations since the 1960s, highlighting the possibility that restriction of genetic diversity might be influential. An alternative explanation may be linked to vitamin D, either due to dietary deficiency (e.g. [100,101,104]), or lighting in the animal enclosures being insufficient to permit normal synthesis [105] (see also Scicluna [45]). Regardless of causation, the persistence of a neonatal skull characteristic exhibited in adult individuals raises potential concern if these captive-bred animals were reintroduced back to the wild.

### 4.4. The potential consequences of captive breeding for reintroduction

This study shows skull morphology and endocranial volume change in a captive-bred carnivorous marsupial. There are several factors that may influence the deviation of skull shape from wild-type, including overall shortening and widening of the skulls in captivity, as seen here. In laboratory colonies, examples of this can include increased behavioural and morphological effects of restricted movement range, homogenous daily diet (differing from what would be consumed in the wild), nutritional deficiencies or the repercussions of stereotypic behaviours (involving repetitive muscle usage) due to their captive environment being unable to fulfil critical factors of an animals’ life history, for example, large home range [80] or a highly specialized diet [81]. By aiming to understand the nature of change in captive populations and the mechanisms causing this, we can address the ways adaptive management of husbandry [106] can help avoid deviation from wild phenotypes, therefore strengthening conservation outcomes.

While evidence for the impacts of captive breeding on skull morphology and brain size is accumulating across many taxa of conservation interest, the consequences of these changes for reintroduced animals or for their offspring are much more speculative [106]. One immediate challenge for captive animals released into the wild, or at least into more complex habitats, is increased cognitive load. In general, wild animals have a greater requirement to process complex information, including among others predator avoidance, foraging and mate choice, whereas the demand for these processes is typically reduced in captivity. Brains are metabolically costly to maintain; therefore, increases in brain size are thought to occur only when it does not risk other aspects of fitness. Thus, limitation of resources is believed to control brain size in wild populations [107]. While not recorded in southern hemisphere species, worthy of mention is Dehnel’s phenomenon [108,109], whereby some small mammals (<100 g; e.g. European moles (*Talpa europaea*) that feature very high metabolic rates and remain active all year) dramatically change skull and brain size (>20%) as an adaptation to harsh changes in climate.

Captivity may result in behavioural or cognitive changes [5,6,10,110], which may be paired with morphological changes that render captive animals less able to manage harder foods in their new environment [27]. Both kinds of changes may alter survival outcomes once captive-bred animals are reintroduced to their wild environment [7].

A common problem with captive breeding programmes is a limited gene pool due to a restricted number of founding individuals, reflecting a subsample of wild diversity. This issue is often accompanied by the inability to increase gene variation due to the low numbers of remaining wild individuals [5]. It is of paramount importance that we understand the effects that captivity has on morphological characteristics, especially if the intention is to reintroduce the animal [7]. If behavioural and phenotypic traits (e.g. particular hunting or foraging behaviours, or a skull shape equipped for a specific diet) are necessary for wild survival, then deviation in captivity and persistence of these observed changes in captive descendants are likely to alter survival outcomes for captive-bred individuals in the case of reintroduction [7,111].

Given that this research examines individuals bred in captivity for substantially more generations than the only previous study on a carnivorous marsupial [23], this advances our understanding of the influence of captivity, in a model species of dasyurid. Significantly, several members of the Dasyuridae family rely on national recovery plans and/or captive breeding for conservation and population recovery (including but not limited to Julia Creek dunnart (*S. douglasi*) [112], Tasmanian devil (*Sarcophilus harrisii*) [113], dibbler (*Parantechinus apicalis*) [114] and chuditch (*Dasyurus geoffroii*)) [115]. While this study highlights an example of laboratory-housed animals (rather than those managed specifically for conservation), these results serve as a valuable opportunity to observe and learn from possible changes that may translate to other dasyurids.

Our results highlight the changes in morphology that can occur in captivity, indicating the importance of maintaining captive diets that align closely with wild diets in both nutrition and ‘toughness’ to avoid morphological changes [14,19,29,37,70–73,116,117]. While laboratory housing is an extreme example, changes documented in these animals can be used to reflect the importance of avoiding deviation from wild phenotypes, particularly if animals are in captivity as part of a conservation programme. Given the relationship between functionality and the morphology of specific skull traits (e.g. masticatory regions), changes to these regions may have substantial adverse effects on captive breeding programmes and reintroduction projects [118]. Understanding the nature of these changes and the mechanisms guiding their differentiation can help us avoid or correct deleterious deviations from wild phenotypes in captive populations using adaptive management of husbandry [106] to promote greater conservation successes.

## Data Availability

Data are accessible at [120].

## Appendix A.

Each morphological measurement was taken, ensuring that the callipers were reset to 0.00 mm between measurements and that the calliper jaws were firmly pressed against each surface. For at least one measurement per skull (randomized), the same measurement was taken several times to ensure it was repeatable and accurate. We found that measurement results very rarely fluctuated between measurement attempts. While very small, the skulls are quite firm; there is no flex, so not much room for movement or change of measurement.

To ensure this was correct, 12 skulls were re-measured to determine PME. Five BL measurements were taken (resetting callipers to 0.00 mm in between each measurement) for 12 skulls. We found that the PME for each measurement was<1%, with the average PME = 0.56%.

See table 3.

**Table 3.** BL measurements of 12 skulls to determine per cent measurement error (PME) = (s.d./mean) × 100.

	BL measurements							
sex + ID	1	2	3	4	5	mean	s.d.	PME
F150	23.38	23.83	23.54	23.59	23.47	23.562	0.169322178	0.718623963
F58	25.05	25.13	25.14	25.47	25.53	25.264	0.219271521	0.867920841
F72	25.12	25.29	25.00	25.12	25.26	25.158	0.117983050	0.468968319
M120	24.31	24.25	24.24	24.49	24.62	24.382	0.166643332	0.683468672
F92	24.04	24.14	24.05	24.10	24.11	24.088	0.042071368	0.174656958
F196	25.03	24.69	25.02	24.96	24.59	24.858	0.203887224	0.820207675
M231	23.95	23.84	23.67	23.66	23.61	23.746	0.143282937	0.603398201
F274	19.98	19.87	19.88	19.90	19.86	19.898	0.048166378	0.242066430
M118	24.77	24.62	24.22	24.54	24.55	24.540	0.201121854	0.819567456
F17	25.62	25.57	25.86	25.79	25.67	25.702	0.120291313	0.468023162
F186	22.79	22.80	22.81	22.91	22.73	22.808	0.064961527	0.284819042
F78	25.04	25.36	25.17	25.31	25.29	25.234	0.128957357	0.511046038
								0.555230563

## Appendix B.

See tables 4 and 5.

**Table 4.** Log–log regressions for eight skull dimensions, and endocranial volume versus body mass for wild-caught fat-tailed dunnarts. *n* = sample size and *r*^2^ = regression coefficient.

	*n*	*r* ^2^	*p*
**BL**	16	0.138	0.156
**ZW**	16	0.455	0.004
**BW**	14	0.445	0.009
**OC**	14	0.086	0.308
**UM**	16	0.012	0.684
**LM**	16	0.016	0.692
**PT**	16	0.199	0.084
**DL**	16	0.267	0.040
**vol**	11	0.395	0.038

**Table 5.** Levene’s tests for homogeneity of variance comparing eight skull dimensions and endocranial volume between captive and wild fat-tailed dunnarts.

	s.d.				
	captive	wild	Levene statistic	*df*	*p*
**BL**	0.7695	1.4980	25.528	1169	<0.001
**ZW**	0.3830	0.7824	22.712	1181	<0.001
**BW**	0.2682	0.3620	5.982	1168	0.015
**OC**	0.2506	0.3027	2.845	1167	0.094
**UM**	0.2443	0.2186	0.234	1186	0.629
**LM**	0.2363	0.2988	6.056	1188	0.015
**PT**	0.3131	0.4423	8.287	1190	0.004
**DL**	0.7008	1.0698	10.715	1191	0.001
**vol**	20.3024	43.8977	39.286	1153	<0.001

## Appendix C.

### Do captive dunnarts have larger endocranial volume simply because they grow to a larger size in captivity?

In this study, the simplest explanation for why the mean endocranial volume was greater in captive dunnarts is that animals in captivity grow to larger mean body size (mass) than their wild counterparts.

To test this, ideally, we would have independent datasets for body mass and endocranial volume in both captive and wild dunnarts, and analysis of covariance could be used to assess differences in endocranial volume between populations while controlling for variation in body mass. However while our datasets included endocranial volumes for 89 captive and 66 wild dunnarts, we have no matching body mass data for the captive individuals, and only 11 body mass records for the wild specimens as recorded on museum labels. This latter dataset allows a first-pass assessment of whether higher endocranial volume in captive dunnarts could be the result of larger body mass. The calculations below assume that the slope and intercept of the relationship between endocranial volume and body size do not vary between captive and wild populations.

To establish the relationship between endocranial volume (*V*_*w*_, in mm^3^) and body mass (*M*_*w*_ in g) for wild dunnarts;

*V*_*w*_ = 348.088 + (7.917 × *M*_*w*_) (*n* = 11, *r*^2^ = 0.458, *p* = 0.022)

— Use this equation to estimate *M*_C-AV_, the estimated mass of a captive dunnart with average endocranial volume. From this study, mean captive volume is 466.2 mm^3^.
  *M*_C-AV_ = (466.15 − 348.088)/7.917
  *M*_C-AV_ = 14.91 g
— Use this equation to estimate *M*_W-AV_, the estimated mass of a wild dunnart with average endocranial volume. From this study, mean wild volume is 448.6 mm^3^
  *M*_W-AV_ = (448.61 − 348.088)/7.917
  *M*_W-AV_ = 12.69 g

Thus, to achieve an increase in endocranial volume of the magnitude documented in this paper (*V*_*C*_ 448.6 to *V*_*W*_ 466.2, 3.9%), captive dunnarts need only be 2.2 g larger on average (*M*_C-AV_ 14.91 – *M*_W-AV_ 12.69) than their wild counterparts.

This seems reasonable. Most literature states fat-tailed dunnarts weigh between 10 and 20 g [38,49,121]. Taking 15 g as the mean weight for wild dunnarts and 16.5 g as the mean live weight of captive animals in the LTU colony (weights of 180 captive-bred animals that were released in a re-introduction trial [104]) gives an increase in body mass in captivity of 1.5 g. So, much of the 3.9% increase in endocranial volume in captive animals could plausibly be due to an overall increase in body size.

## References

[1] Fraser DJ. 2008 How well can captive breeding programs conserve biodiversity? A review of salmonids. Evol. Appl. **1**, 535–586. (10.1111/j.1752-4571.2008.00036.x) 25567798 PMC3352391

[2] Hogg CJ, Ivy JA, Srb C, Hockley J, Lees C, Hibbard C, Jones M. 2015 Influence of genetic provenance and birth origin on productivity of the Tasmanian devil insurance population. Conserv. Genet. **16**, 1465–1473. (10.1007/s10592-015-0754-9)

[3] Blanchet S, Páez DJ, Bernatchez L, Dodson JJ. 2008 An integrated comparison of captive-bred and wild Atlantic salmon (Salmo salar): implications for supportive breeding programs. Biol. Conserv. **141**, 1989–1999. (10.1016/j.biocon.2008.05.014)

[4] Snyder NFR, Derrickson SR, Beissinger SR, Wiley JW, Smith TB, Toone WD, Miller B. 1996 Limitations of captive breeding in endangered species recovery. Conserv. Biol. **10**, 338–348. (10.1046/j.1523-1739.1996.10020338.x)

[5] Lacy RC, Honer BE. 1996 Effects of inbreeding on skeletal development of Rattus villosissimus. J. Hered. **87**, 277–287. (10.1093/oxfordjournals.jhered.a023001) 8776876

[6] Price EO. 2002 Animal domestication and behavior. New York, NY: CABI Publishing.

[7] O’regan HJ, Kitchener AC. 2005 The effects of captivity on the morphology of captive, domesticated and feral mammals. Mammal Rev. **35**, 215–230. (10.1111/j.1365-2907.2005.00070.x)

[8] Moseby K, Carthey AJR, Parkhurst T. 2015 The influence of predators and prey naivety on reintroduction; Current and future directions. In Advances in reintroduction biology of australian and new zealand fauna (eds EDP Armstrong, MW Hayward, D Moro, PJ Seddon), pp. 29–42. Clayton, MO: CSIRO Publishing.

[9] Carlstead K, Shepherdson DJ. 1994 Effects of environmental enrichment on reproduction. Zoo Biol. **13**, 447–458. (10.1002/zoo.1430130507)

[10] Price EO. 1999 Behavioral development in animals undergoing domestication. Appl. Anim. Behav. Sci. **65**, 245–271. (10.1016/s0168-1591(99)00087-8)

[11] Dunston EJ *et al*. 2017 Does captivity influence territorial and hunting behaviour? Assessment for an ex situ reintroduction program of African lions Panthera leo. Mammal Rev. **47**, 254–260. (10.1111/mam.12101)

[12] McDougall PT, Réale D, Sol D, Reader SM. 2006 Wildlife conservation and animal temperament: causes and consequences of evolutionary change for captive, reintroduced, and wild populations. Anim. Conserv. **9**, 39–48. (10.1111/j.1469-1795.2005.00004.x)

[13] Stoinski TS, Beck BB. 2004 Changes in locomotor and foraging skills in captive-born, reintroduced golden lion tamarins (Leontopithecus rosalia rosalia). Am. J. Primatol. **62**, 1–13. (10.1002/ajp.20002) 14752809

[14] Hartstone-Rose A, Selvey H, Villari JR, Atwell M, Schmidt T. 2014 The three-dimensional morphological effects of captivity. PLoS One **9**, e113437. (10.1371/journal.pone.0113437) 25409498 PMC4237414

[15] Stojanovic D, Neeman T, Hogg CJ, Everaardt A, Wicker L, Young CM, Alves F, Magrath MJL, Heinsohn R. 2021 Differences in wing shape of captive, critically endangered, migratory orange-bellied Parrot Neophema chrysogaster relative to wild conspecifics. Emu Austral Ornithol. **121**, 178–186. (10.1080/01584197.2021.1872389)

[16] Siciliano-Martina L, Light JE, Lawing AM. 2021 Cranial morphology of captive mammals: a meta-analysis. Front. Zool. **18**, 1–13. (10.1186/s12983-021-00386-0) 33485360 PMC7825229

[17] Siciliano-Martina L, Light JE, Lawing AM. 2021 Changes in canid cranial morphology induced by captivity and conservation implications. Biol. Conserv. **257**, 109143. (10.1016/j.biocon.2021.109143)

[18] Siciliano‐Martina L, Light JE, Riley DG, Lawing AM. 2022 One of these wolves is not like the other: morphological effects and conservation implications of captivity in Mexican wolves. Anim. Conserv. **25**, 77–90. (10.1111/acv.12724)

[19] Siciliano-Martina L, Michaud M, Tanis BP, Scicluna EL, Lawing AM. 2022 Endocranial volume increases across captive generations in the endangered Mexican wolf. Sci. Rep. **12**, 1–8. (10.1038/s41598-022-12371-6) 35581330 PMC9114419

[20] Lewton KL. 2017 The effects of captive versus wild rearing environments on long bone articular surfaces in common chimpanzees (Pan troglodytes). PeerJ **5**, e3668. (10.7717/peerj.3668) 28828263 PMC5560229

[21] Pelletier M, Niinimäki S, Salmi AK. 2021 Influence of captivity and selection on limb long bone cross-sectional morphology of reindeer. J. Morphol. **282**, 1533–1556. (10.1002/jmor.21403) 34323317

[22] Courtney Jones SK, Munn AJ, Byrne PG. 2018 Effect of captivity on morphology: negligible changes in external morphology mask significant changes in internal morphology. R. Soc. Open Sci. **5**, 172470. (10.1098/rsos.172470) 29892434 PMC5990819

[23] Guay PJ, Parrott M, Selwood L. 2012 Captive breeding does not alter brain volume in a marsupial over a few generations. Zoo Biol. **31**, 82–86. (10.1002/zoo.20393) 23900892

[24] Wisely SM, Ososky JJ, Buskirk SW. 2002 Morphological changes to black-footed ferrets (Mustela nigripes) resulting from captivity. Can. J. Zool. **80**, 1562–1568. (10.1139/z02-160)

[25] Antonelli T, Leischner CL, Hartstone-Rose A. 2022 The cranial morphology of the black-footed ferret: a comparison of wild and captive specimens. Animals **12**, 2708. (10.3390/ani12192708) 36230449 PMC9558532

[26] Katz DC, Grote MN, Weaver TD. 2017 Changes in human skull morphology across the agricultural transition are consistent with softer diets in preindustrial farming groups. Proc. Natl Acad. Sci. USA **114**, 9050–9055. (10.1073/pnas.1702586114) 28739900 PMC5576786

[27] Mitchell DR, Wroe S, Ravosa MJ, Menegaz RA. 2021 More challenging diets sustain feeding performance: applications toward the captive rearing of wildlife. Integr. Org. Biol. **3**, 1–13. (10.1093/iob/obab030) PMC8653637 34888486

[28] Cooper DM, Yamaguchi N, Macdonald DW, Patterson BD, Salkina GP, Yudin VG, Dugmore AJ, Kitchener AC. 2023 Getting to the meat of it: the effects of a captive diet upon the skull morphology of the lion and tiger. Animals **13**, 3616. (10.3390/ani13233616) 38066967 PMC10705091

[29] Hanegraef H, Spoor F. 2024 Maxillary morphology of chimpanzees: captive versus wild environments. J. Anat. **244**, 977–994. (10.1111/joa.14016) 38293709 PMC11095307

[30] Balcarcel AM, Geiger M, Clauss M, Sánchez‐Villagra MR. 2021 The mammalian brain under domestication: discovering patterns after a century of old and new analyses. J. Exp. Zool. Part B **338**, 460–483. (10.1002/jez.b.23105) PMC9787656 34813150

[31] Ramírez Rozzi FV, González-José R, Pucciarelli HM. 2005 Cranial growth in normal and low-protein-fed saimiri. an environmental heterochrony. J. Hum. Evol. **49**, 515–535. (10.1016/j.jhevol.2005.06.002) 16051314

[32] Kruska D. 1996 The effect of domestication on brain size and composition in the mink (Mustela vison). J. Zool. **239**, 645–661. (10.1111/j.1469-7998.1996.tb05468.x)

[33] Burns JG, Saravanan A, Helen Rodd F. 2009 Rearing environment affects the brain size of guppies: lab‐reared guppies have smaller brains than wild‐caught guppies. Ethology **115**, 122–133. (10.1111/j.1439-0310.2008.01585.x)

[34] Wisely SM, Santymire RM, Livieri TM, Marinari PE, Kreeger JS, Wildt DE, Howard J. 2005 Environment influences morphology and development for in situ and ex situ populations of the black-footed ferret (Mustela nigripes). Anim. Conserv. **8**, 321–328. (10.1017/S1367943005002283)

[35] Curtis AA, Orke M, Tetradis S, van Valkenburgh B. 2018 Diet-related differences in craniodental morphology between captive-reared and wild coyotes, Canis latrans (Carnivora: Canidae). Biol. J. Linn. Soc. **123**, 677–693. (10.1093/biolinnean/blx161)

[36] Cox PG, Morris PJR, Hennekam JJ, Kitchener AC. 2020 Morphological and functional variation between isolated populations of British red squirrels (Sciurus vulgaris). J. Zool. **312**, 271–283. (10.1111/jzo.12829)

[37] Zuccarelli MD. 2004 Research Article: comparative morphometric analysis of captive vs. wild African lion (Panthera leo) skulls. Bios. **75**, 131–138. (10.1893/0005-3155(2004)0752.0.co;2)

[38] Morton S. 1978 An ecological study of sminthopsis crassicaudata (Marsupialia: Dasyuridae) I. Distribution, study areas and methods. Wildl. Res. **5**, 151–162. (10.1071/WR9780151)

[39] Morton S. 1978 An ecological study of Sminthopsis carasicaudata (Marsupialia: Dasyuridae) III. Reproduction and life history. Wildl. Res. **5**, 183–211. (10.1071/WR9780183)

[40] Morton SR, Denny MJS, Read DG. 1983 Habitat preferences and diets of sympatric Sminthopsis crassicaudata and S. macroura (Marsupialia: Dasyuridae). Aust. Mammal. **6**, 29–34. (10.1071/AM83003)

[41] Morton SR. 1995 Fat-tailed dunnart *Sminthopsis crassicaudata* (Gould, 1844). In ‘The mammals of australia’ (ed. ER Strahan), pp. 129–131. Sydney, Australia: Reed Books.

[42] Cooper SJB, Adams M, Labrinidis A. 2000 Phylogeography of the Australian dunnart Sminthopsis crassicaudata (Marsupialia: Dasyuridae). Aust. J. Zool. **48**, 461–473. (10.1071/ZO00014)

[43] Department of Energy, Environment and Climate Action (DEECA). 2023 Flora and Fauna Guarantee Act threatened list. Melbourne, Australia: Victorian State Government.

[44] Scicluna EL, Gill BP, Robert KA. 2021 Fat-tailed dunnarts (Sminthopsis crassicaudata) of the Werribee grasslands: a case study of a species in decline. Aust. J. Zool. **69**, 27–32. (10.1071/ZO21014)

[45] Scicluna E. 2023 Fat-tailed dunnarts (Sminthopsis crassicaudata): conservation, phenotypic change in captivity and relevance to reintroduction outcomes. Doctoral dissertation, La Trobe University, Bundoora, Victoria, Australia.

[46] Martin P. 1965 The potentialities of the fat-tailed marsupial mouse, Sminthopsis crassicaudata (Gould), as a laboratory animal. Aust. J. Zool. **13**, 559–562. (10.1071/ZO9650559)

[47] Breed WG, Leigh CM, Bennett JH. 1989 Sperm morphology and storage in the female reproductive tract of the fat‐tailed dunnart, Sminthopsis crassicaudata (Marsupialia: Dasyuridae). Gamete Res. **23**, 61–75. (10.1002/mrd.1120230107) 2744705

[48] Newton AH *et al*. 2024 Embryology of the fat-tailed dunnart (Sminthopsis crassicaudata): a marsupial model for comparative mammalian developmental and evolutionary biology. Dev. Dyn. **254**, 142–157. (10.1002/dvdy.711) 38721717 PMC11809135

[49] Bennett JH, Breed WG, Hayman DL, Hope RM. 1990 Reproductive and genetic studies with a laboratory colony of the Dasyurid marsupial Sminthopsis crassicaudata. Aust. J. Zool. **37**, 207–222. (10.1071/ZO9890207)

[50] Hall ER, Russell WC. 1933 Dermestid beetles as an aid in cleaning bones. J. Mammal. **14**, 372–374. (10.1093/jmammal/14.4.372)

[51] Woolley PA. 1994 The dasyurid marsupials of New Guinea: use of museum specimens to assess seasonality of breeding. Sci. N. Guin. **20**, 49–55.

[52] Guay PJ, Iwaniuk AN. 2008 Captive breeding reduces brain size in waterfowl (Anseriformes). Condor **110**, 276–284. (10.1525/cond.2008.8424)

[53] Logan CJ, Clutton-Brock TH. 2013 Validating methods for estimating endocranial volume in individual red deer (Cervus elaphus). Behav. Process. **92**, 143–146. (10.1016/j.beproc.2012.10.015) 23137587

[54] Iwaniuk AN. 2001 Interspecific variation in sexual dimorphism in brain size in nearctic ground squirrels (Spermophilus spp.). Can. J. Zool. **79**, 759–765. (10.1139/cjz-79-5-759)

[55] Marino L. 1999 Brain growth in the harbor porpoise and pacific white-sided dolphin. J. Mammal. **80**, 1353–1360. (10.2307/1383186)

[56] Cottingham KL, Lennon JT, Brown BL. 2005 Knowing when to draw the line: designing more informative ecological experiments. Front. Ecol. Environ. **3**, 145–152. (10.1890/1540-9295(2005)003[0145:kwtdtl]2.0.co;2)

[57] D’Antoni AV, Donaldson OI, Schmidt C, Macchi V, De Caro R, Oskouian RJ, Loukas M, Shane Tubbs R. 2017 A comprehensive review of the anterior fontanelle: embryology, anatomy, and clinical considerations. Childs. Nerv. Syst. **33**, 909–914. (10.1007/s00381-017-3406-1) 28396968

[58] Gould SJ. 1966 Allometry and size in ontogeny and phylogeny. Biol. Rev. **41**, 587–638. (10.1111/j.1469-185x.1966.tb01624.x) 5342162

[59] dos Reis SF, Pessôa LM, Strauss RE. 1990 Application of size-free canonical discriminant analysis to studies of geographic differentiation. Braz. J. Genet. **13**, 509–520.

[60] Wobbrock JO, Findlater L, Gergle D, Higgins JJ. 2011 The aligned rank transform for nonparametric factorial analyses using only anova procedures. In Proceedings of the SIGCHI conference on human factors in computing systems, pp. 143–146. New York, NY: Association for Computing Machinery. (10.1145/1978942.1978963)

[61] Kay M, Elkin L, Higgins J, Wobbrock J. 2021 ARTool: aligned rank transform for nonparametric factorial ANOVAs. R package version 0.11, 1(10.5281). See https://cran.r-project.org/web//packages/ARTool/ARTool.pdf.

[62] R Core Team. 2023 R: A language and environment for statistical computing. Vienna, Austria: R Foundation for Statistical Computing. See https://www.R-project.org/.

[63] Jackson S. 2007 Australian mammals: biology and captive management. Clayton, Australia: CSIRO Publishing.

[64] Scicluna EL, Newton AH, Hutchison JC, Dimovski AM, Fanson KV, D’Souza G, Whitehead S, Pask AJ. 2025 Breeding fat-tailed dunnarts (Sminthopsis crassicaudata) in captivity: Revised practices to minimize stress whilst maintaining considerations of wild biology. Dev. Dyn. **254**, 189–204. (10.1002/dvdy.755) 39895010 PMC11809136

[65] King J, Glover R. 1945 The relative effects of dietary constituents and other factor upon calculus formation and gingival disease in the ferret. J. Pathol. Bacteriol. **57**, 353–362.

[66] Haberstroh LI, Ullrey DE, Sikarski JG, Richter NA, Colmery BH, Myers TD. 1984 Diet and oral health in captive amur tigers (Panthera tigris altaica). J. Zoo Anim. Med. **15**, 142–146. (10.2307/20094710)

[67] Gawor JP, Reiter AM, Jodkowska K, Kurski G, Wojtacki MP, Kurek A. 2006 Influence of diet on oral health in cats and dogs. J. Nutr. **136**, 2021–2023. (10.1093/jn/136.7.2021S) 16772485

[68] Morton S. 1978 An ecological study of sminthopsis crassicaudata (Marsupialia: Dasyuridae) II. Behaviour and social organization. Wildl. Res. **5**, 163–182. (10.1071/WR9780163)

[69] van der Velden U. 1984 Effect of age on the periodontium. J. Clin. Periodontol. **11**, 281–294. (10.1111/j.1600-051x.1984.tb01325.x) 6371061

[70] Hollister N. 1917 Some effects of environment and habit on captive lions. Proc. U. S. Natl. Mus. **53**, 177–193. (10.5479/si.00963801.53-2196.177)

[71] Hollister N. 1918 East African mammals in the United States National Museum, part 1: Insectivora, Chiroptera, and Carnivora. Bull. US. Natl. Mus. **99**, 1–194. (10.5479/si.03629236.99.1)

[72] Yamaguchi N, Kitchener AC, Gilissen E, Macdonald DW. 2009 Brain size of the lion (Panthera leo) and the tiger (P. tigris): implications for intrageneric phylogeny, intraspecific differences and the effects of captivity. Biol. J. Linn. Soc. **98**, 85–93. (10.1111/j.1095-8312.2009.01249.x)

[73] Saragusty J, Shavit-Meyrav A, Yamaguchi N, Nadler R, Bdolah-Abram T, Gibeon L, Hildebrandt TB, Shamir MH. 2014 Comparative skull analysis suggests species-specific captivity-related malformation in lions (Panthera leo). PLoS One **9**, e94527. (10.1371/journal.pone.0094527) 24718586 PMC3981823

[74] Neaux D *et al*. 2021 How changes in functional demands associated with captivity affect the skull shape of a wild boar (Sus scrofa). Evol. Biol. **48**, 27–40. (10.1007/s11692-020-09521-x)

[75] Grossnickle DM. 2020 Feeding ecology has a stronger evolutionary influence on functional morphology than on body mass in mammals. Evolution **77**, 610–628. (10.1111/evo.13929) 31967667

[76] Cordy DR. 1957 Osteodystrophia fibrosa accompanied by visceral accumulation of lead. Cornell Vet. **47**, 480–490. 13473362

[77] Caligiuri R, Kollias GV, Spencer C. 1989 Metabolic bone disease in dromedary camels (Camelus dromedarius). J. Zoo Wildl. Med. **20**, 482–487.

[78] Junge RE, Gannon FH, Porton I, McAlister WH, Whyte MP. 2000 Management and prevention of vitamin D deficiency rickets in captive-born juvenile chimpanzees (Pan troglodytes). J. Zoo Wildl. Med. **31**, 361–369. (10.1638/1042-7260(2000)031[0361:MAPOVD]2.0.CO;2) 11237144

[79] Mason GJ. 1991 Stereotypies: a critical review. Anim. Behav. **41**, 1015–1037. (10.1016/s0003-3472(05)80640-2)

[80] Kroshko J, Clubb R, Harper L, Mellor E, Moehrenschlager A, Mason G. 2016 Stereotypic route tracing in captive carnivora is predicted by species-typical home range sizes and hunting styles. Anim. Behav. **117**, 197–209. (10.1016/j.anbehav.2016.05.010)

[81] Lyons J, Young RJ, Deag JM. 1997 The effects of physical characteristics of the environment and feeding regime on the behavior of captive felids. Zoo Biol. **16**, 71–83. (10.1002/(sici)1098-2361(1997)16:13.3.co;2-j)

[82] Mifsud G. 1994 Aspects of behaviour of the Julia Creek dunnart. BSc thesis, LaTrobe University, Bundoora, Victoria, Australia.

[83] Churchill S. 2001 The ecology of the sandhill dunnart, Sminthopsis psammophila. Report to the Department for Environment and Heritage, South Australian Government.

[84] Laidlaw WS, Hutchings S, Newell GR. 1996 Home range and movement patterns of Sminthopsis leucopus (Marsupialia: Dasyuridae) in coastal dry Heathland, Anglesea, Victoria. Aust. Mammal **19**, 1–9. (10.1071/AM96001)

[85] Kruska D. 1988 Mammalian domestication and its effect on brain structure and behavior. In Intelligence and evolutionary biology (eds HJ Jerison, I Jerison), pp. 211–250. Berlin, Heidelberg: Springer. (10.1007/978-3-642-70877-0_13)

[86] Tamlin AL, Bowman J, Hackett DF. 2009 Separating wild from domestic American mink Neovison vison based on skull morphometries. Wildl. Biol. **15**, 266–277. (10.2981/08-004)

[87] Kruska DCT, Steffen K. 2013 Comparative allometric investigations on the skulls of wild cavies (Cavia aperea) versus domesticated guinea pigs (C. aperea f. porcellus) with comments on the domestication of this species. Mamm. Biol. **78**, 178–186. (10.1016/j.mambio.2012.07.002)

[88] Walsh RE, Aprígio Assis AP, Patton JL, Marroig G, Dawson TE, Lacey EA. 2016 Morphological and dietary responses of chipmunks to a century of climate change. Glob. Chang. Biol. **22**, 3233–3252. (10.1111/gcb.13216) 26732228

[89] Fisher DO, Dickman CR. 1993 Diets of insectivorous marsupials in arid Australia: selection for prey type, size or hardness? J. Arid Environ. **25**, 397–410. (10.1006/jare.1993.1072)

[90] Gardner SL, Anderson S. 2001 Persistent fontanelles in rodent skulls. Am. Mus. Novit. **2001**, 1–16. (10.1206/0003-0082(2001)3272.0.co;2)

[91] Wiig Ø. 1985 Multivariate variation in feral American mink (Mustela vison) from Southern Norway. J. Zool. **206**, 441–452. (10.1111/j.1469-7998.1985.tb05669.x)

[92] Bennett CV, Goswami A. 2013 Statistical support for the hypothesis of developmental constraint in marsupial skull evolution. BMC Biol. **11**, 1–14. (10.1186/1741-7007-11-52) 23622087 PMC3660189

[93] Cook LE, Newton AH, Hipsley CA, Pask AJ. 2021 Postnatal development in a marsupial model, the fat-tailed dunnart (Sminthopsis crassicaudata; Dasyuromorphia: Dasyuridae). Commun. Biol. **4**, 1028. (10.1038/s42003-021-02506-2) 34475507 PMC8413461

[94] Gemmell RT, Johnston G, Bryden MM. 1988 Osteogenesis in two marsupial species, the bandicoot Isoodon macrourus and the possum Trichosurus vulpecula. J. Anat. **159**, 155–164. 3248963 PMC1262018

[95] Nesslinger CL. 1956 Ossification centers and skeletal development in the postnatal virginia opossum. J. Mammal. **37**, 382–394. (10.2307/1376739)

[96] Baker AM, Mutton TY, Hines HB. 2013 A new dasyurid marsupial from Kroombit tops, south-east Queensland, Australia: the Silver-headed Antechinus, Antechinus argentus sp. nov. (Marsupialia: Dasyuridae). Zootaxa **3746**, 201–239. (10.11646/zootaxa.3746.2.1) 25113476

[97] Davies DP, Ansari BM, Cooke TJ. 1975 Anterior fontanelle size in the neonate. Arch. Dis. Child. **50**, 81–83. (10.1136/adc.50.1.81) 1124948 PMC1544472

[98] DelRosso LM, Gonzalez-Toledo E, Hoque R. 2013 A three-month-old achondroplastic baby with both obstructive apneas and central apneas. J. Clin. Sleep Med. **09**, 287–289. (10.5664/jcsm.2504) PMC3578685 23495340

[99] Tucker J, Choudhary AK, Piatt J. 2016 Macrocephaly in infancy: benign enlargement of the subarachnoid spaces and subdural collections. J. Neurosurg **18**, 16–20. (10.3171/2015.12.PEDS15600) 26942270

[100] Steyn M, Meiring JH, Nienaber WC, Loots M. 2002 Large fontanelles in an early 20th century rural population from South Africa. Int. J. Osteoarchaeol. **12**, 291–296. (10.1002/oa.628)

[101] Kiesler J, Ricer R. 2003 The abnormal fontanel. Am. Fam. Physician **67**, 2547–2552. 12825844

[102] Paladini D, Sglavo G, Penner I, Pastore G, Nappi C. 2007 Fetuses with down syndrome have an enlarged anterior fontanelle in the second trimester of pregnancy. Ultrasound Obstet. Gynecol. **30**, 824–829. (10.1002/uog.5129) 17803259

[103] Seymour AM, Montgomery ME, Costello BH, Ihle S, Johnsson G, St. John B, Taggart D, Houlden BA. 2001 High effective inbreeding coefficients correlate with morphological abnormalities in populations of South Australian koalas (Phascolarctos cinereus). Anim. Conserv. **5**, 211–219. (10.1017/S1367943001001251)

[104] Bass MH, Fisch GR. 1961 Increased intracranial pressure with bulging fontanel. a symptom of vitamin a deficiency in infants. Neurology **11**, 1091–1094. (10.1212/wnl.11.12.1091) 13865618

[105] Kennel KA, Drake MT, Hurley DL. 2010 Vitamin D deficiency in adults: when to test and how to treat. Mayo Clin. Proc. **85**, 752–758. (10.4065/mcp.2010.0138) 20675513 PMC2912737

[106] Crates R, Stojanovic D, Heinsohn R. 2023 The phenotypic costs of captivity. Biol. Rev. Camb. Philos. Soc. **98**, 434–449. (10.1111/brv.12913) 36341701

[107] Gonda A, Herczeg G, Merilä J. 2013 Evolutionary ecology of intraspecific brain size variation: a review. Ecol. Evol. **3**, 2751–2764. (10.1002/ece3.627) 24567837 PMC3930043

[108] Dehnel A. 1949 Studies on the genus Sorex L. Annales Universitatis Mariae Curie-Skłodowska **4**, 18–102.

[109] Nováková L, Lázaro J, Muturi M, Dullin C, Dechmann DKN. 2022 Winter conditions, not resource availability alone, may drive reversible seasonal skull size changes in moles. R. Soc. Open Sci. **9**, 220652. (10.1098/rsos.220652) 36133148 PMC9449468

[110] Price EO. 1984 Behavioral aspects of animal domestication. Q. Rev. Biol. **59**, 1–32. (10.1086/413673)

[111] Tarszisz E, Dickman CR, Munn AJ. 2014 Physiology in conservation translocations. Conserv. Physiol. **2**, 1–19. (10.1093/conphys/cou054) PMC4732500 27293675

[112] Department of Environment and Resource Management (DERM). 2009 National recovery plan for the julia creek dunnart (sminthopsis douglasi). Report to the department of the environment, water, heritage and the arts, canberra. Brisbane, Queensland, Australian: Queensland Parks and Wildlife Service.

[113] Department of Primary Industries, Parks, Water and Environment (DPIPWE). 2010 Recovery plan for the tasmanian devil (sarcophilus harrisii). Hobart, Australia: Department of Primary Industries, Parks, Water and Environment.

[114] Department of conservation and land management (DCLM). 2003 Dibbler recovery plan. Perth, Australia: Department of Conservation and Land Management.

[115] Department of Environment and Conservation (DEC). 2012 Chuditch (Dasyurus geoffroii) recovery plan. Wildlife management program no. 54. Perth, Australia: Department of Environment and Conservation.

[116] Howell AB. 1925 Pathologic skulls of captive lions. J. Mammology **6**, 163–168.

[117] Antonelli T. 2006 The cranial morphology of the black-footed ferret: a comparison of wild and captive specimens. MS thesis, University of South Carolina, Columbia, SC. 10.3390/ani12192708 PMC9558532 36230449

[118] O’Regan HJ, Kitchener AC. 2005 The effects of captivity on the morphology of captive, domesticated and feral mammals. Mamm. Rev. **35**, 215–230. (10.1111/j.1365-2907.2005.00070.x)

[119] Sikes RS, Animal Care and Use Committee of the American Society of Mammalogists. 2016 2016 Guidelines of the American Society of Mammalogists for the use of wild mammals in research and education. J. Mammal. **97**, 663–688. (10.1093/jmammal/gyw078) 29692469 PMC5909806

[120] Scicluna E. 2025 Fat-tailed dunnart skull morphology [Dataset]. Dryad Digital Repository. (10.5061/dryad.prr4xgxvr)

[121] Menkhorst P, Knight F. 2001 Field guide to the mammals of Australia Oxford, UK: Oxford University Press.

